# EHMTI-0183. “Maternal smoking during pregnancy and chronic headache at school children in Uzbekistan.”

**DOI:** 10.1186/1129-2377-15-S1-B7

**Published:** 2014-09-18

**Authors:** S Gazieva, Anna Prokhorova, N Rashidova

**Affiliations:** 1 Neurology, Tashkent Medical Academy, Tashkent, Uzbekistan

## Introduction

Current tobacco smoking has been associated with headache. However it is notknown if maternal smoking during pregnancy is associated with headache in theiroffspring.

## Aim

The aim of thisresearch was evaluated the association between maternal smoking duringpregnancy with headache in school-aged children.

## Methods

Twoprospective cohorts of 101 children aged 9-11 years from Tashkent (Uzbekistan)and 95 children aged 7-8 years from Samarkand (Uzbekistan) were studied. Dataon maternal smoking were collected at birth and data on headache and other confoundingfactors were collected at school age using the same questionnaire completed bythe mothers in the two cities. Primary headache was defined as a motherreporting that his/her child had ≥ 2 episodes of headache in the two lastweeks, without any associated organic symptoms.

## Results

Prevalence of headache was reported by the mother was 35% in Tashkent and 21%in Samarkand. After adjustment, children whose mothers smoked >=8 cigarettesper day during the pregnancy presented higher prevalence of primary headachethan their counterparts in both cohorts.

## Conclusions

Inspite of adjusting for a long list of potential confounders, maternal smokingduring pregnancy was associated with headache in 7-11 years olds in two Uzbekistancities of contrasting economic wealth.

No conflict of interest.

